# Dysphagia Aortica With Deglutition Syncope in an Elderly Woman

**DOI:** 10.14309/crj.0000000000002146

**Published:** 2026-05-28

**Authors:** Prakash Thomas, Brenton G. Davis, Mitchell L. Liverant

**Affiliations:** 1 Department of Internal Medicine, Kaiser Permanente Santa Clara Medical Center, Santa Clara, CA; 2 Division of Gastroenterology, Kaiser Permanente San Francisco Medical Center, San Francisco, CA; 3 Division of Gastroenterology, Kaiser Permanente Oakland Medical Center, Oakland, CA

## CASE REPORT

**Figure 1.**
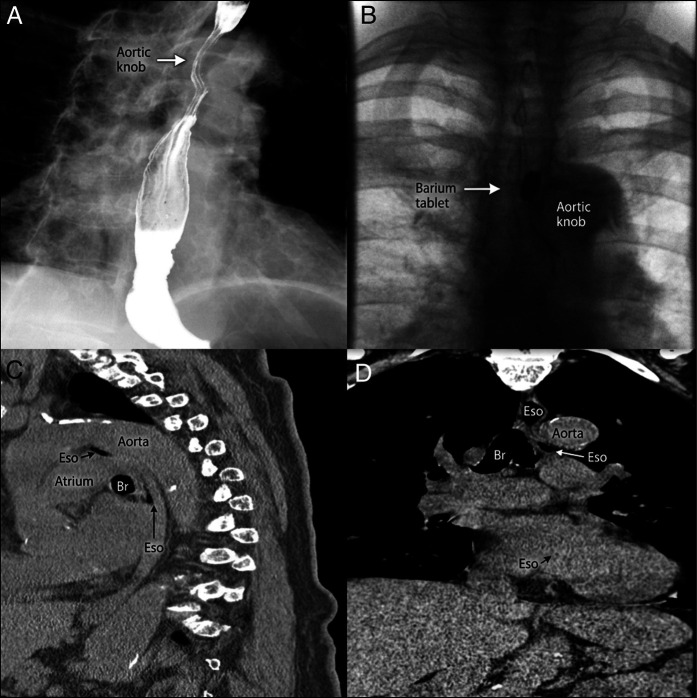
Dysphagia aortica on barium esophagram and CT. (A) Prominent aortic impression on the mid-thoracic esophagus. (B) 12.5 mm tablet transiently retained at the impression (arrow). (C) Sagittal CT showing esophageal indentation by the thoracic aorta. (D) Axial CT demonstrating focal esophageal narrowing adjacent to the aorta. CT, computed tomography.

An 83-year-old woman experienced recurrent syncope within the first 1–2 bites of solid food, with 4 episodes in 1 year, preceded by nausea and emesis. Electrocardiogram, troponins, and a 30-day monitor were unrevealing. She denied baseline odynophagia, weight loss, or previous neurological events. Barium esophagram with tablet challenge showed a prominent aortic impression on the mid-thoracic esophagus with transient retention of a 12.5 mm tablet.^1,2^ (Figure 1) Contrast-enhanced chest computed tomography confirmed focal esophageal compression between an ectatic, atherosclerotic thoracic aorta and the vertebral column, accentuated by kyphosis.^1,2^ (Figure 1) Mechanical distension from food boluses at the compressed segment triggers a vasovagal reflex causing deglutition syncope.^3^ Given the diagnosis and its low utility, endoscopy was deferred.^1,2^ Behavioral modification, small bites, thorough chewing, and liquids with solids improved symptoms at 8-month follow-up.

## DISCLOSURES

Author contributions: P. Thomas: literature review; drafting of the article; final approval. BG Davis: supervision; critical revision for important intellectual content; final approval. ML Liverant: clinical care and data acquisition; critical revision; final approval. P. Thomas is the article guarantor.

Financial disclosure: None to report.

Previous presentation: Presented as an abstract at the American College of Gastroenterology Annual Scientific Meeting, October 24–29, 2025; Phoenix, Arizona.

Informed consent was obtained for this case report.
